# Changes in Primary and Secondary Metabolite Levels in Response to Gene Targeting-Mediated Site-Directed Mutagenesis of the Anthranilate Synthase Gene in Rice

**DOI:** 10.3390/metabo2041123

**Published:** 2012-12-18

**Authors:** Hiroaki Saika, Akira Oikawa, Ryo Nakabayashi, Fumio Matsuda, Kazuki Saito, Seiichi Toki

**Affiliations:** 1 Plant Genome Engineering Research Unit, Agrogenomics Research Center, National Institute of Agrobiological Sciences, Tsukuba, Ibaraki 305-8602, Japan; E-Mail: saika@affrc.go.jp; 2 Riken Plant Science Center, Yokohama, Kanagawa 230-0045, Japan; E-Mails: oikawa@psc.riken.jp (A.O.); ryona@psc.riken.jp (R.N.); fmatsuda@ruby.kobe-u.ac.jp (F.M.); ksaito@faculty.chiba-u.jp (K.S.); 3 Organization of Advanced Sciences and Technology, Kobe University, Kobe, Hyogo 657-8501, Japan; 4 Graduate School of Pharmaceutical Sciences, Chiba University, Inage-ku, Chiba 263-8522, Japan; 5 Kihara Institute of Biological Research, Yokohama City University, Yokohama, Kanagawa 244–0813, Japan

**Keywords:** gene targeting, indole alkaloid, metabolic engineering, rice, tryptophan

## Abstract

Gene targeting (GT) via homologous recombination allows precise modification of a target gene of interest. In a previous study, we successfully used GT to produce rice plants accumulating high levels of free tryptophan (Trp) in mature seeds and young leaves via targeted modification of a gene encoding anthranilate synthase—a key enzyme of Trp biosynthesis. Here, we performed metabolome analysis in the leaves and mature seeds of GT plants. Of 72 metabolites detected in both organs, a total of 13, including Trp, involved in amino acid metabolism, accumulated to levels >1.5-fold higher than controls in both leaves and mature seeds of GT plants. Surprisingly, the contents of certain metabolites valuable for both humans and livestock, such as γ-aminobutyric acid and vitamin B, were significantly increased in mature seeds of GT plants. Moreover, untargeted analysis using LC-MS revealed that secondary metabolites, including an indole alkaloid, 2-[2-hydroxy-3-β-*D*-glucopyranosyloxy-1-(1*H*-indol-3-yl)propyl] tryptophan, also accumulate to higher levels in GT plants. Some of these metabolite changes in plants produced via GT are similar to those observed in plants over expressing mutated genes, thus demonstrating that *in vivo* protein engineering via GT can be an effective approach to metabolic engineering in crops.

## 1. Introduction

Higher plants use a myriad of metabolites to adapt to environmental change. Some of these are valuable for human and livestock as nutritional, insecticidal and pharmaceutical bioactives. The development of plant biotechnology enables us to produce value-added plants in which a specific useful metabolite accumulates to a high level. Metabolic pathway engineering via conventional transformation approaches has been especially successful in the over expression of genes encoding enzymes in biosynthetic pathways and the knock-down of genes encoding enzymes in catabolic pathways [1]. For example, provitamin A was fortified successfully by introduction of the β-carotene biosynthetic pathway into rice grains [2].

**Figure 1:**
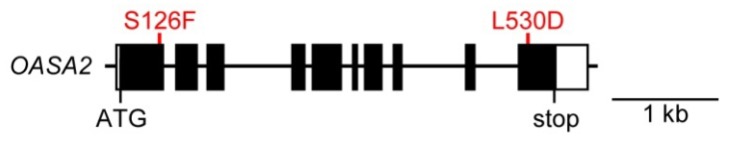
Structure of the OASA2 gene. The mutations S126F (S [TCC] to F [TTC] at amino acid 126) and L530D (L [CTT] to D [GAC] at amino acid 530) were introduced successfully into the endogenous OASA2 gene via gene targeting (GT) [4]. Molecular analysis revealed that true GT, in which the wild-type OASA2 gene was mutated as expected, had occurred successfully [4].

Tryptophan (Trp) plays an important role in human and livestock nutrition and is one of the limiting essential amino acids in cereals. Thus, Trp fortification is one of the breeding aims in cereals, including rice. The level of Trp in plants is strictly controlled by feedback inhibition of the activity of anthranilate synthase (AS)—a key enzyme of Trp biosynthesis. The AS enzyme consists of two sets of α and β subunits. The α subunits catalyze the amination of chorismate and the removal of the enolpyruvyl side chain and possess a binding site for Trp for feedback inhibition. In rice, there are two isoforms of the α subunit of AS: OASA1 and OASA2 (Figure 1, [3]). On the other hand, the β subunits function as a glutamine amidotransferase to transfer an amido group from glutamine to the α subunits.

To date, Trp fortification in rice has been achieved by conventional mutation approaches, by screening of rice mutants that showed insensitivity to 5-methyltryptophan (5MT)—a Trp analogue [5,6,7,8]. Missense mutations in the OASA1 gene were discovered in some of these mutants, suggesting that these mutations might affect Trp accumulation [8]. Trp fortification in rice has also been achieved by the overexpression of mutated enzyme genes. Trp accumulates in calli, young leaves and mature seeds of transgenic rice overexpressing OASA1D—an OASA1 gene with a Trp-insensitive mutation (D323N)—under the control of a strong constitutive promoter [3,9,10]. Increased contents of free amino acids other than Trp and secondary metabolites involved in Trp metabolism have also been reported in the leaves and seeds of OASA1D overexpressing plants [9,10]. Moreover, untargeted metabolome analysis revealed that secondary metabolites, including an indole alkaloid, also accumulate to higher levels in the leaves and calli of OASA1D overexpressing plants [11,12].

Kinetic analysis with reconstituted AS isozymes showed that OASA2 was more sensitive to Trp than OASA1 [13]. OASA2 catalytic activity is assumed to be negligible at the endogenous Trp concentration found in rice leaves [13]. Moreover, the OASA2 mRNA level is higher than that of OASA1 in leaves and roots [3]. This suggested that accumulation of free Trp in rice could be achieved more easily by modification of the enzymatic properties of OASA2, rather than OASA1. Structure-based protein engineering of OASA2 showed that S126F and L530D mutations confer Trp insensitivity and enhance the catalytic activity of AS, respectively [14]. It has also been shown that free Trp accumulates to high levels in transformed rice calli overexpressing a modified OASA2 gene harboring S126F/L530D mutations [14].

Precise modification of a targeted gene via homologous-recombination-mediated gene targeting (GT) is a powerful tool not only for the analysis of the gene function of interest, but also for the molecular breeding of crops, including rice. The first reliable report of GT in rice described disruption of the Waxy gene that encodes granule-bound starch synthase [15]. In this latter report, GT cells were obtained using positive and negative selection markers to select cells in which transformation events had occurred successfully and to stop growth of cells in which the donor DNA had integrated at random, respectively. To date, successful knock-out and knock-in of targeted genes via GT using a similar selection system has been reported in several loci in the rice genome [15,16,17,18,19,20]. On the other hand, we succeeded in producing novel herbicide-tolerant rice plants containing point mutations in the acetolactate synthase (ALS) gene—a major target of many herbicides—by site-directed mutagenesis via GT [21]. Site-directed mutagenesis via GT can be considered a “clean” transformation technology, since no exogenous positive selection marker is used. Using a similar strategy, we succeeded in the production of Trp-fortified rice by the introduction of S126F/L530D mutations into the OASA2 gene via GT [4].

In this study, we performed metabolome analysis using capillary electrophoresis—mass spectrometry (CE-MS) and liquid chromatography—mass spectrometry (LC-MS) in GT plants harboring S126F/L530D mutations in the OASA2 gene. The results suggest that the levels of many metabolites were altered (mainly increased) and that mutations in the OASA2 gene affected various metabolic pathways.

## 2. Results

### 2.1. 5MT Sensitivity of GT Plants

**Figure 2:**
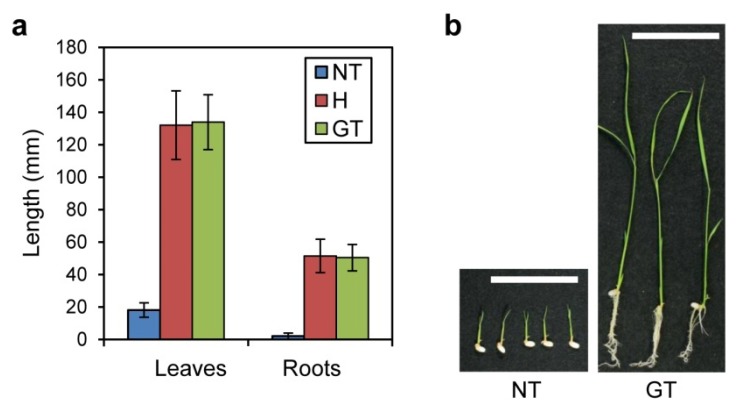
Sensitivity of non-transformant and GT plants to 5MT. Plants were grown in the presence of 250 μM 5MT for 10 days.(**a**) Length of leaves and roots in non-transformants (NT) and plants homozygous (GT) and heterozygous (H) for the modified OASA2 gene in the T_2_ generation. Values are average ± SD (NT *n* = 9, H *n* = 14, GT *n* = 8).(**b**) Photographs of 10-day-old NT and GT plants grown under 250 μM 5MT conditions. Bars = 5 cm.

In previous studies, 5MT-resistant mutant rice plants have been produced by somaclonal mutation, chemical mutagenesis and γ-ray irradiation [5,6,7,8]. However, there are no reports of rice mutants harboring OASA2 with S126F/L530D mutations having been screened successfully by conventional mutation approaches, probably because the chances of two kinds of mutations that improve enzymatic properties occurring simultaneously in a single gene are quite low. Here, we compared sensitivity to the Trp analogue 5MT between non-transformed and our high Trp-accumulating GT plants (hereafter GT plants). The leaves and roots of homozygous and heterozygous GT plants were shown to be insensitive to 5MT compared to non-transformants, as expected (Figure 2). These results suggest that, theoretically, rice mutants harboring OASA2 with S126F/L530D mutations produced by conventional mutation approaches could be screened with 5MT.

### 2.2. Primary and Secondary Metabolites Involved in Amino Acid Metabolism in GT Plants

**Figure 3:**
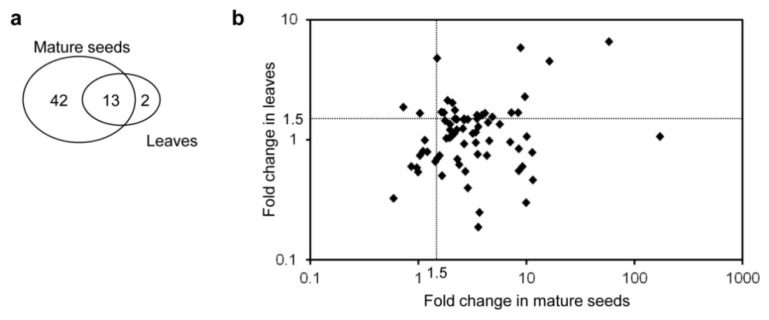
Changes in primary and secondary metabolite levels in mature seeds and leaves. (**a**) Venn diagram of metabolites with >1.5-fold higher intensity in mature seeds and seedlings in GT plants compared to non-transformants. (**b**) Comparison of the fold change in metabolites in mature seeds and leaves. Fold change is presented as the ratio of the intensity of each metabolite in GT plants homozygous for mutated OASA2 compared to non-transformants. There is a weak positive relationship between the fold change in metabolites in mature seeds and that in leaves (*R* = 0.18). Metabolites are listed in Supplementary Table S2 and Supplementary Table S3.

The contents of free amino acids other than Trp in mature seeds of homozygous GT plants were much higher than in seeds of non-transformants [4]. Similar results have been reported in the seeds of OASA1D overexpressing plants grown under field conditions [9]. We assumed that the contents of primary and secondary metabolites involved in amino acid metabolism have changed. To research this, levels of primary and secondary metabolites in leaves and mature seeds of GT plants were analyzed using CE-MS. A total of 72 metabolites was detected in all extracts prepared from leaves and mature seeds of non-transformant and GT plants. We analyzed the fold change presented as the ratio of the peak intensity of GT plants to that of non-transformants. Peak intensity is presented as the ratio of the peak area of each metabolite to that of internal standard. Of these 72 metabolites, 55 and 15 were increased significantly (5% level of *t*-test; >1.5-fold higher) in GT plants compared to non-transformants in mature seeds and leaves, respectively, and a total of 13 metabolites was increased significantly by >1.5-fold in both mature seeds and leaves (Figure 3 and Table 1). Of the 20 free amino acids, eight (including Trp) accumulated >1.5-fold higher in both leaves and mature seeds (Table 1). Trp was increased most, as noted in our previous report, and in OASA1D overexpressing plants (Supplementary Table S1) [4,9]. These results suggested that Trp accumulates specifically in plants harboring the α subunit of AS with Trp-insensitive mutations together with unknown mechanisms by which free amino acid synthesis and/or accumulation is enhanced in response to Trp hyper-accumulation, which can regulate the levels of metabolites in these plants [4,9,22]. By contrast, only one metabolite, glutathione-SH (GSH), was significantly decreased (>1.5-fold lower) in GT plants compared to non-transformants in both mature seeds and leaves. 

**Figure 4:**
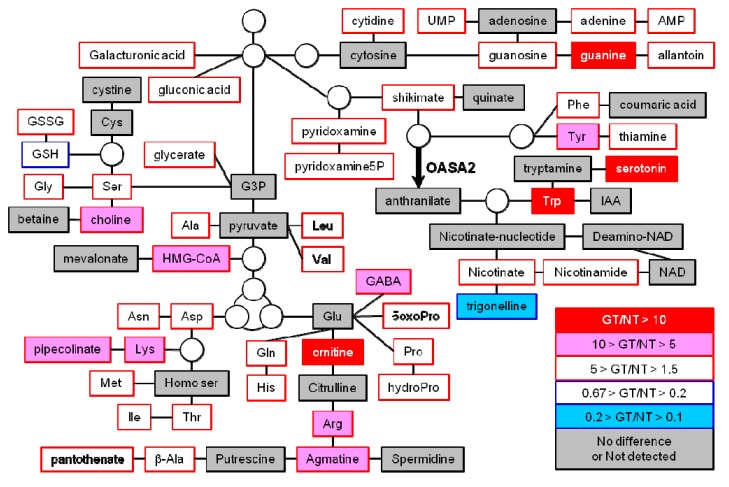
Changes in metabolite levels derived from amino acids in mature seeds. Mature seeds of non-transformant (NT) and homozygous GT plants (GT) were subjected to metabolome analysis using CE-MS (see Tables S1–S3). Representative metabolites are shown in this pathway map, and the fold change in metabolite levels in mature seeds of GT plants to that in NT is shown using a color scale, as indicated.

We focused on changes in metabolites in mature seeds as the edible part of the plants and because more types of metabolites showed large changes compared to leaves (Figure 3b). Levels of 50 of the 70 metabolites successfully annotated exhibited significant differences between non-transformants and GT plants (Figure 4 and Tables S1–S3). We identified the following notable features: (1) the levels of almost all annotated metabolites increased in the mature seeds of GT plants; (2) the levels of Trp and its derived metabolite, serotonin (5-hydroxytryptamine), were greatly increased; and (3) considering those metabolites that were increased in both mature seeds and seedlings, Glu-derived amino acids and Glu-derived metabolites, including ornithine and agmatine, increased to a high level (Figure 4, Table 1 and Table S1 to S3).

**Table 1:** Primary and secondary metabolites whose levels were significantly increased (>1.5-fold higher) in both mature seeds and leaves in GT plants compared to non-transformants.

**metabolites**	**Mature seeds**							**Leaves**						
	NT^1^			GT^1^			Fold change^2^	NT^1^			GT^1^			Fold change^2^
Arg	0.702	±	0.316	5.802	±	1.985	8.27	17.506	±	5.638	29.345	±	5.385	1.68
Asn	4.249	±	2.034	11.190	±	0.740	2.63	34.053	±	8.110	51.133	±	11.145	1.50
Ile	0.158	±	0.036	0.342	±	0.038	2.16	6.838	±	1.382	12.030	±	3.354	1.76
Leu	0.156	±	0.047	0.320	±	0.032	2.06	4.613	±	0.861	9.353	±	2.143	2.03
Lys	0.154	±	0.060	1.471	±	0.654	9.55	4.848	±	1.581	11.085	±	2.262	2.29
Met	0.05	±	0.021	0.080	±	0.033	1.61	0.575	±	0.257	0.980	±	0.453	1.71
Trp	0.456	±	0.275	26.286	±	16.105	57.59	5.270	±	1.563	34.481	±	4.896	6.54
Val	0.414	±	0.071	1.434	±	0.284	3.47	12.244	±	1.805	19.670	±	4.225	1.61
Ornithine	0.002	±	0.003	0.034	±	0.013	16.16	0.248	±	0.114	1.116	±	0.492	4.50
Agmatine	0.001	±	0.002	0.011	±	0.005	8.82	0.021	±	0.010	0.123	±	0.050	5.84
Pyridoxamine	0.002	±	0.002	0.008	±	0.003	4.10	0.015	±	0.002	0.025	±	0.008	1.67
Thiamine	0.054	±	0.006	0.092	±	0.012	1.71	0.128	±	0.021	0.214	±	0.037	1.68
5-OxoPro	0.047	±	0.011	0.087	±	0.024	1.85	0.264	±	0.152	0.559	±	0.247	2.12

^1^ Signal intensity presented as mean ± SD (*n* = 6). NT; non-transformant, GT; GT plants homozygous for mutated OASA2. ^2^ Fold change is presented as the ratio of the intensity of each metabolite in GT compared to NT.

### 2.3. Untargeted LC-MS Metabolic Profiling Analysis in GT Plants

**Figure 5:**
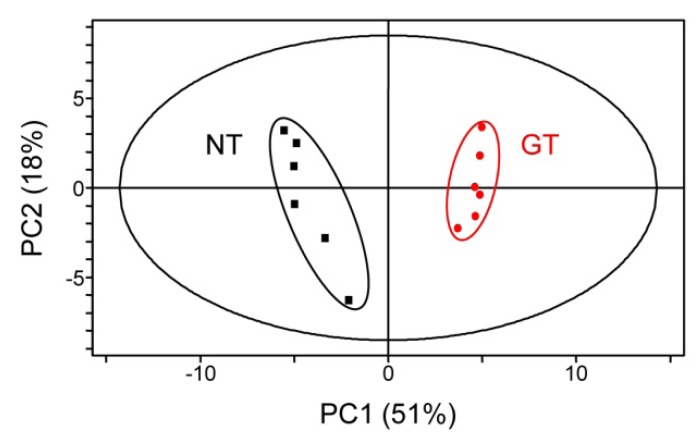
Principal component analysis (PCA) of untargeted negatively charged metabolic profile. PCA was performed using Simca-P11. Black squares and red circles represent samples obtained from mature seeds of NT and GT, respectively.

Considering that Trp and serotonin accumulate in mature seeds of GT plants (Figure 4), the levels of Trp-derived secondary metabolites, including indole alkaloids, are also possibly altered. To confirm this, we performed an untargeted metabolic profiling analysis using LC-MS. Examination of metabolic profile data obtained from LC-MS by principal component analysis (PCA) showed that the plot representing untargeted negatively charged metabolic profile obtained from mature seeds was divided into two clusters in a space defined by the axes of two components, demonstrating that there is an effect of S126F/L530D mutations in the OASA2 gene on the metabolic profiles (Figure 5). Similar results were obtained in PCA analysis using the untargeted positively charged metabolic profile obtained from mature seeds (data not shown).

A total of 577, 1,141 and 580 peaks were detected in extracts prepared from mature seeds, leaves and hulls of mature seeds, respectively. In mature seeds, leaves and hulls, the number of peaks in which the change was >10-fold higher is much greater than the number in which the change is >10-fold lower (Supplementary Figure S1). These results suggest that activation of the Trp synthetic pathway and/or Trp accumulation may result in the general enhancement of core metabolic pathways, including those of amino acid metabolism, and that many metabolites accumulate to higher levels in the mature seeds of GT plants (Figure 4 and Tables S2–S5).

**Figure 6:**
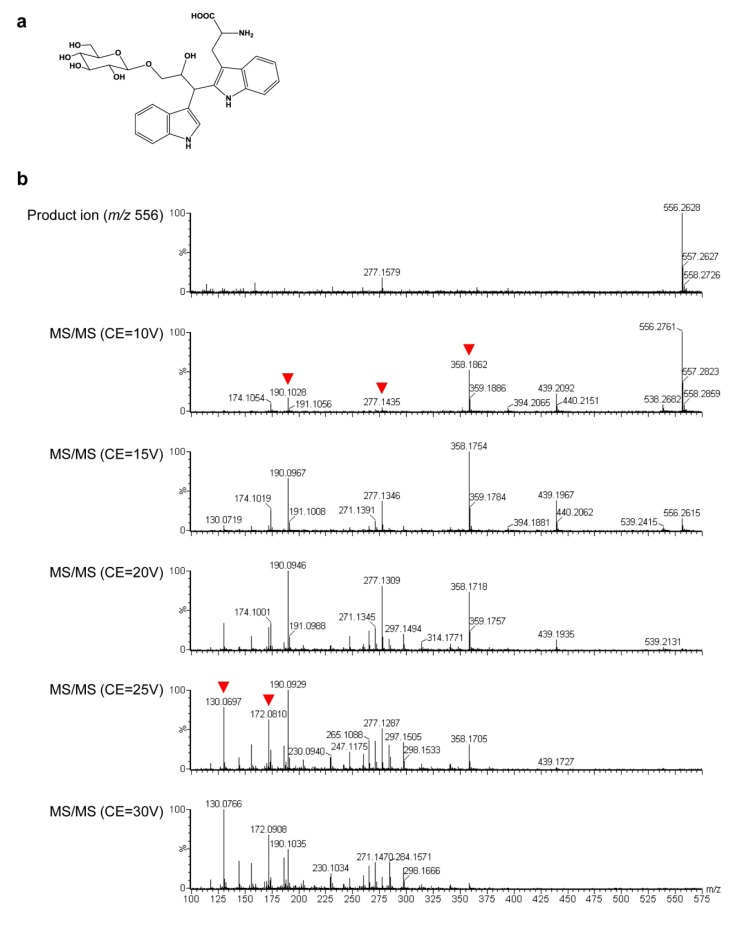
An indole alkaloid present in increased amount in mature seeds of GT plants. (**a**) Structure of the indole alkaloid, 2-[2-hydroxy-3-β-D-glucopyranosyloxy-1-(1H-indol-3-yl)propyl]-tryptophan. (**b**) MS/MS spectra of peak #4673. Red arrowheads show the fragments corresponding to the fragments shown in a previous report [12].

A previous study found that an indole alkaloid, 2-[2-hydroxy-3-β-D-glucopyranosyloxy-1-(1H-indol-3-yl)propyl]tryptophan (MW 555, Figure 6a), accumulated to levels 170- and 50-fold higher, respectively, in leaf sheath and the first expanded leaf of OASA1D overexpressing rice seedlings than those of non-transformants [12]. Moreover, this indole alkaloid was reported to accumulate in the callus of OASA1D overexpressing rice and in a phenylalanine- and Trp-accumulating rice mutant [11,23]. These results suggest that this indole alkaloid might also accumulate in our GT plants. To confirm this, we focused on peaks #4022 (Table S4) and #4673 (Table S5). The chemical component of the peak was analyzed structurally by LC-MS/MS and deduced to be this same indole alkaloid (Figure 6b). Moreover, peaks deduced to correspond to this indole alkaloid were detected in leaves and hulls, and their intensities in GT plants were much higher than those of non-transformants (data not shown).

## 3. Discussion

Indole-acetic acid (IAA) was also not detected in mature seeds of non-transformants, but was detected in those of GT plants. In the calli and seeds of OASA1D overexpressing plants, free IAA content was reported to be 1.8-fold to 1.9-fold higher and 2.6-fold to 57-fold higher compared to non-transformants, respectively [10,11]. As a natural plant hormone, IAA—the main auxin in plants—plays an important role in plant growth and development. Morphological alterations have not been reported in OASA1D overexpressing and GT plants [4,9]. However, IAA may affect other agronomical traits, so further analysis may be required.

Five metabolites, including anthranilate, were unaffected in both mature seeds and leaves (Table S3). Anthranilate levels were also shown to be unaffected in OASA1D overexpressing calli [3]; however, its level was highly increased in leaves of OASA1D overexpressing plants [9]. In these plants, OASA1D expression is under the control of the strong constitutive maize ubiquitin promoter [3]. The mRNA levels of OASA1 and OASA2 genes were increased under 5MT treatment in the 5MT tolerant mutant, although they were decreased in wild-type [27]. In the previous report, rice calli overexpressing wild-type OASA2 gene showed 5MT tolerant [3]. These results suggested that expression levels of OASA1 and OASA2 genes affect the sensitivity to 5MT. On the other hand, it was shown that mRNA levels of the mutated OASA2 in GT plants were the same as those in non-transformants, suggesting that OASA2 transcription in GT plants is controlled in the same manner as endogenous OASA2 [4]. According to the Rice Expression Profile Database (RiceXPro) [28,29], OASA2 expression levels in leaves are relatively low. Thus, the difference in anthranilate levels may be due to the expression levels of OASA1D and OASA2 harboring S126F/L530D.

Surprisingly, some metabolites, including γ-aminobutyric acid (GABA)—a major inhibitory neurotransmitter in mammals—and vitamin B (thiamine, nicotinamide, pantothenate and pyridoxamine) were increased significantly in mature seeds and/or leaves (Figure 4 and Table 1, S2 and S3), suggesting that our GT plants are a nutrient-rich food and feed. On the other hand, the indole alkaloid, deduced to be 2-[2-hydroxy-3-β-D-glucopyranosyloxy-1-(1*H*-indol-3-yl)propyl]-tryptophan, was increased in mature seeds, leaves and hull (Figure 6 and Table S4 and Table S5, data not shown). This indole alkaloid was also detected in the roots of OASA1D overexpressing plants, although the increase in this case was not significant [12]. The functions of this alkaloid are still unknown, although it is thought to accumulate in rice to some extent. The body weight gain and feed intake of chickens fed OASA1D overexpressing rice and non-transformed rice supplemented with crystalline Trp were higher than those of chickens fed non-transformed rice alone [30]. These results suggest that feeding of OASA2 GT plants might enhance chicken growth and that this indole alkaloid might not be toxic to livestock and humans.

It was reported that the contents of free amino acids, including Trp, were increased in seeds of OASA1D overexpressing plants grown under field conditions compared to non-transformants, suggesting the possibility that Trp accumulation affects the expression levels and patterns of genes that encode enzymes involved in amino acid metabolism [9]. This alteration of gene expression could also occur in GT plants. Recent metabolome analysis of a mutant rice lacking OsGS1;1, which encodes cytosolic glutamine synthetase, showed a mutant-specific correlation between tryptamine and other primary metabolites, such as phenylalanine, tyrosine, glucose-6-phosphate and fructose-6-phosphate [31]. Moreover, an imbalance in the levels of sugars, amino acids and metabolites involved in the TCA cycle in OsGS1;1 mutant was reported [31]. These findings suggested the possibility that modification of the Trp pathway in GT plants affects not only nitrogen metabolism, but also carbon metabolites. Taken together, we assume that the contents of various kinds of metabolites were changed (mainly increased) in GT plants as follows. Trp accumulation or an imbalance of free amino acids composition could modify the gene expression of enzymes involved in free amino acid metabolism, so that the levels of free amino acids would increase. This increment of free amino acids enhances the accumulation of nitrogen-source metabolites, as shown in Figure 4. It also affects carbon-source metabolites involved in glycolysis and the TCA cycle. Consequently, a global change of nitrogen and carbon metabolites occurs. In this study, leaves grown on rich medium containing Murashige and Skoog basal salts [32] and sucrose were used for metabolome analysis. Thus, it is very important and interesting to analyze the changes in metabolite levels, including carbon-source metabolites, involved in glycolysis, the TCA cycle and the photosynthesis of GT plants grown under the medium containing minimum nitrogen- and/or carbon sources. The further metabolome analysis could afford new insights into any global metabolic imbalances caused by metabolic engineering of the Trp biosynthetic pathway.

Metabolic engineering by conventional transformation approaches has also been reported. For example, high levels accumulation of GABA were achieved successfully by the overexpression of a truncated OsGAD2 gene that encodes glutamate decarboxylase lacking the strong autoinhibitory domain present at the C-terminus [33]. GABA-fortified rice seeds are expected to be useful in the control and/or cure of hypertension in humans [34]. Moreover, lysine—a limiting essential amino acid in rice grains—can be accumulated by the knock-down of two transcriptional factors RISBZ1/RPBF that regulate expression of the OsLKR/SDH gene encoding lysine catabolizing enzyme [35]. It is theoretically possible that useful plants could be produced by deletion of the C-terminal domain in OsGAD2 and the simultaneous introduction of some mutations into the promoter of the OsLKR/SDH, gene based on conventional mutagenesis approaches. However, these approaches are thought not to be viable at present because the frequencies are quite low. On the other hand, site-directed mutagenesis via GT can be used to produce such plants successfully at much higher frequencies, as shown by our OsALS and OASA2 GT plants [4,21]. In fact, we showed that mutant plants similar to our GT plant, but produced by conventional mutagenesis approaches, can be screened with 5MT (Figure 2). Moreover, unlike conventional transgenic approaches, with site-directed mutagenesis via GT, the wild-type gene can be eliminated completely. In our previous report, the herbicide-tolerance level of GT homozygous plants containing point mutations in OsALS gene exceeded that of plants over expressing the mutated OsALS gene, suggesting that complete elimination of the wild-type ALS enzyme that shows higher herbicide sensitivity enables the breeder to confer quite higher herbicide-tolerance on rice plants [21]. Thus, the complete substitution of a wild-type gene by GT has been shown to be efficacious in producing novel plant phenotypes that surpass conventional transformation approaches [21]. Thus, we believe that precise modification via GT will be of increasing significance in the metabolic engineering of crops.

## 4. Experimental Section

### 4.1. Plant Materials

Untransformed rice (cv. Nipponbare) and GT rice harboring the OASA2 gene with S126F/L530D mutations (#3-4 in [4]) were used in this study.

Seeds of plants grown in the greenhouse were stored at room temperature with low humidity for 1 year. Seeds of non-transformant and homozygous GT seeds were dehulled, and hulls and de-hulled seeds (mature seeds) were sampled for metabolome analysis.

The progenies of heterozygous T_2_ plants were grown on Murashige and Skoog medium without phytohormones [32,36] for 2 weeks. Leaves (the aerial part of the seedlings) and roots were harvested separately and frozen immediately in liquid N_2_. Genotyping analysis was performed as follows. We successfully introduced silent mutations (added a *Hin*dIII site 16-bp upstream of S126F; CAGCTT to AAGCTT, deleted an *Eco*RV site 22-bp upstream of L530D; GATATC to GATTTC) in addition to the S126F and L530D mutations in the endogenous OASA2 gene via GT [4]. PCR was performed using genomic DNA extracted from roots as a template and primer sets located between S126F and the additional *Hin*dIII site (5'-GAAGTGGAGTTGACGAGCTGTGGAAAAA-3' and 5'-TAAGAATCGATAAGGGAGCAGATTGGAAGG-3'). PCR products digested with *Hin*dIII were separated on an agarose gel for genotyping of individuals. Two bands (0.4 and 0.1 kb) and three bands (0.5, 0.4 and 0.1 kb) were detected in homozygous and heterozygous GT plants, respectively. Leaves of non-transformant and homozygous GT plants were sampled for metabolome analysis.

### 4.2. 5MT Sensitivity Test

Heterozygous GT seeds of the T_2_ generation were inoculated on Murashige and Skoog medium [32,36] with 250 μM 5MT solidified with 0.4% gelrite after sterilization and cultured at 30 °C under constant light. Leaf and root lengths were measured after 10 days. After taking these morphometeric measurements, the leaf tissues were harvested and subjected to genotyping analysis as described above.

### 4.3. Metabolome Analysis Using CE-MS and Measurement of Amino Acid Contents

Sample preparation and amino acid quantification by CE-TOFMS basically followed the method of Ohkama-Ohtsu [37].

Rice samples were homogenized with zirconia beads in a Mixer Mill (Retsch, Haan, Germany) at 27 Hz for 3 min. Twenty volumes of methanol (20 μL/mg fresh weight), including 8 μM of internal standards (200 μM of methionine sulfone for cation analyses and 200 μM of camphor 10-sulfonic acid for anion analyses) that was used for compensation of the peak area after CE-MS analysis were added, and again homogenized at 27 Hz for 1 min. The sample solution was then centrifuged at 20,400 g for 3 min at 4 °C. 500 μL of chloroform and 200 μL of water were added to the supernatants. This mixture was mixed on a vortex mixer for 3 min and centrifuged at 20,400 g for 3 min at 4 °C. The upper layer was evaporated for 30 min at 45 °C by a centrifugal concentrator and then separated into two layers. The upper layer (100–200 μL) was filtered centrifugally through a Millipore 5-kDa cutoff filter at 9,100 g for 150 min. The filtrate was dried for 120 min in a centrifugal concentrator. The residue was dissolved in 20 μL of water. This solution was used for CE-MS analyses. All CE-TOFMS experiments were performed using an Agilent CE capillary electrophoresis system (Agilent Technologies, Waldbronn, Germany), an Agilent G3250AA LC/MSD TOF system (Agilent Technologies, Palo Alto, CA, USA), an Agilent 1100 series binary HPLC pump and the G1603A Agilent CE-MS adapter and G1607A Agilent CE-ESI-MS sprayer kit. The G2201AA Agilent ChemStation software for CE and the Analyst QS software for TOFMS were used. Separations were carried out using a fused silica capillary (50 μM i.d. × 100 cm total length) filled with 1 M formic acid for cation analyses or with 20 mM ammonium formate (pH 10.0) for anion analyses as the electrolyte. The sample solutions were injected at 50 mbar for 15 sec (15 nL). Prior to each run, the capillary was flushed with electrolyte for 5 min. The applied voltage was set at 30 kV. The capillary temperature was maintained at 20 °C, and the sample tray was cooled below 4 °C. Fifty percent (v/v) methanol/water containing 0.5 μM reserpine was delivered as the sheath liquid at 10 μL/min. ESI-TOFMS was conducted in the positive ion mode for cation analyses or in the negative ion mode for anion analyses, and the capillary voltage was set at 4 kV. A flow rate of heated dry nitrogen gas (heater temperature 300 °C) was maintained at 10 psig. In TOFMS, the fragmentor, skimmer and Oct RFV voltage were set at 110 V, 50 V and 160 V for cation analyses or at 120 V, 60 V and 220 V for anion analyses, respectively. Automatic recalibration of each acquired spectrum was performed using reference masses of reference standards. The methanol dimer ion ([2M+H]^+^, *m/z* = 65.0597) and reserpine ([M+H]^+^, *m/z* = 609.2806) for cation analyses or the formic acid dimer ion ([2M-H]^¯^, *m/z* = 91.0037) and reserpine ([M-H]^¯^, *m/z* = 607.2661) for anion analyses provided the lock mass for exact mass measurements. Exact mass data were acquired at a rate of 1.5 cycles/s over a 50–1,000 *m/z* range.

### 4.4. Untargeted Metabolome Analysis Using LC-MS

Hulls and seedlings were homogenized in five volumes of 80% MeOH, including 0.5 mg/L of lidocaine and 0.5 mg/L of 10-camphorsulphonic acid. Extraction was performed by a mixer mill (MM300, Retsch) with zirconia beads for 10 min at 20 Hz. After centrifugation at 15,000 g, the extracts were applied to an Oasis HLB μ-elution plate (Waters) equilibrated with 80% MeOH, including 0.1% acetic acid for filtration. Mature seeds were extracted with ten volumes of 80% MeOH using a mixer mill (MM300, Retsch) at 20 Hz for 10 min. After centrifugation for 10 min at 15,000 *g*, 500 μL of the supernatant were transferred into a tube and diluted in 0.1% acetic acid. The extract solutions were applied to an Oasis HLB μ-elution plate (Waters) equilibrated with the solvent for filtration. The extracts were evaporated by SPD2010 SpeedVac and then dissolved in 200 μL of water containing five internal standards as follows; 0.5 mg/L oflidocain, 1.0 mg/L ampiciline, 1.0 mg/L torperizone, 0.5 mg/L 10-camphorsulfonic acid and 1.0 mg/L 2-naphthalene-4-sodium sulfate.

The extracts (3 μL) were applied to an LC-MS system with an electrospray ionization (ESI) interface (LC, Waters Aquity UPLC system; MS, Waters Q-Tof Premier). On the LC system, the following conditions were set: column, Aquity bridged ethylhybrid (BEH) C18 (poresize, 1.7 μM, length 2.1 μ 100 mm, Waters); column oven temperature, 38 °C; flow rate, 0.3 mL min^−1^; solvent, solvent A (H_2_O with 0.1% formic acid) and solvent B (CH_3_CN with 0.1% formic acid); gradient profile, 0 min 0% B, 15.5 min 100% B ,17.0 min 100% B, 17.1 min 0% B, 20.0 min 0% B. The MS and MS/MS conditions and data processing were as described previously [38].

## 5. Conclusions

We demonstrate that *in vivo* enzyme engineering via GT is an effective approach to metabolic engineering in crops. Since site-directed mutagenesis via GT can introduce desirable mutations into target enzyme with various types of mutations as expected, GT technology could be a useful tool to produce plants in the field of both molecular analysis of metabolic pathway and molecular breeding.

## Supplementary Files

Supplementary File 1 Supplementary (PDF, 166 KB)
