# Supplementary material for: Changes in Primary and Secondary Metabolite Levels in Response to Gene Targeting-Mediated Site-Directed Mutagenesis of the Anthranilate Synthase Gene in Rice
Source: Metabolites. 2012 Dec 18;2(4):1123–38. doi: 10.3390/metabo2041123 (PMC3901229; doi:10.3390/metabo2041123)
Supplement: Supplementary File 1 — Supplementary (PDF, 166 KB) [file metabolites-02-01123-s001.pdf]

Supplementry:

Figure S1. The classification of fold change in signal intensities of peaks detected in extracts prepared from mature seeds, leaves and hulls using LC-MS.

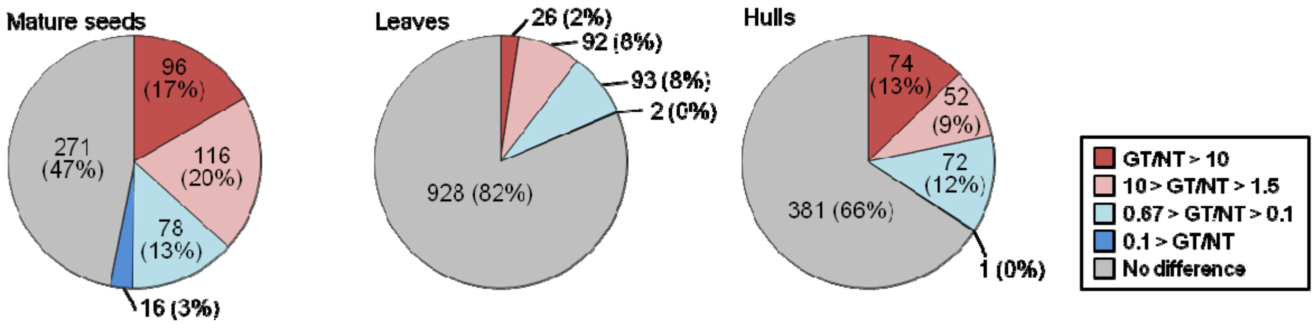

Table S1. Changes in free amino acid content in mature seeds determined by CE-MS.

Amino acids	NT (nmol/g)[1]			GT (nmol/g)[1]			Fold change[2]	*P* value[3]
Ala	298.4	±	89.8	999.4	±	251.9	3.35	**
Arg	63.3	±	28.5	523.2	±	179.0	8.27	**
Asn	846.3	±	405.1	2228.9	±	147.5	2.63	**
Asp	767.1	±	339.9	1192.0	±	182.4	1.55	*
Cys		ND		148.4	±	124.5	-	-
Gln	59.0	±	61.0	131.8	±	27.7	2.23	*
Glu	1294.3	±	130.5	1101.0	±	221.0	0.85	
Gly	63.9	±	35.4	112.3	±	11.5	1.76	**
His	104.5	±	57.8	235.8	±	77.3	2.26	**
Ile	11.8	±	2.6	25.5	±	2.8	2.16	**
Leu	11.9	±	3.6	24.5	±	2.4	2.06	**
Lys	19.9	±	7.7	190.5	±	84.7	9.55	**
Met	7.7	±	3.2	12.4	±	5.2	1.61	*
Phe	9.0	±	2.4	40.4	±	8.0	4.47	**
Pro	311.7	±	186.0	599.5	±	199.5	1.92	*
Ser	133.5	±	21.6	284.7	±	32.8	2.13	**
Thr	48.4	±	17.5	96.7	±	26.2	2.00	**
Trp	49.4	±	29.8	2842.1	±	1741.3	57.59	**
Tyr	30.9	±	6.8	173.6	±	28.8	5.61	**
Val	38.3	±	6.6	132.9	±	26.3	3.47	**

[1]Values are mean ± SD (n = 6). ND: not-detected. NT: non-transformant. GT: GT plants homozygous for mutated OASA2. [2]Fold change is presented as the ratio of the content of each amino acid in mature seeds of GT to those of NT. [3]Asterisks (* and **) indicate significant differences between NT and GT at $P < 0.05$ and $P < 0.01$, respectively, as determined by t-test.

Table S2. Changes in anion metabolite levels in mature seeds determined by CE-MS.

Annotation Name	Ave m/z	Signal intensity[1]						Fold change[1]	*P* value[2]
		NT			GT				
4-Coumarate	163.0395	0.003	±	0.001	0.002	±	0.001	0.76	
Quinate	191.0555	0.006	±	0.000	0.006	±	0.001	0.98	
Lactate	89.02482	0.131	±	0.041	0.135	±	0.037	1.03	
Benzoate	121.0295	0.048	±	0.007	0.052	±	0.006	1.10	
2-Furoate	111.0091	0.000	±	0.001	0.000	±	0.001	1.10	
5-Oxohexanoate	129.0557	0.003	±	0.001	0.004	±	0.001	1.16	
4-Pyridoxate	182.0453	0.002	±	0.001	0.003	±	0.001	1.19	
4-Oxovaleric acid	115.0399	0.002	±	0.001	0.003	±	0.002	1.20	
Deamino-NAD	663.0874	0.000	±	0.000	0.000	±	0.001	1.27	
NAD	662.1028	0.003	±	0.001	0.005	±	0.001	1.42	**
Pantothenate	218.1025	0.019	±	0.007	0.032	±	0.009	1.65	*
5-OxoPro	128.0352	0.047	±	0.011	0.087	±	0.024	1.85	**
T6P	421.0743	0.020	±	0.006	0.039	±	0.005	1.94	**
AMP	346.0547	0.037	±	0.014	0.071	±	0.012	1.94	**
F6P;G1P	259.0207	0.003	±	0.002	0.007	±	0.002	1.98	**
Raffinose	503.1614	0.007	±	0.004	0.017	±	0.006	2.27	**
Shikimate	173.045	0.001	±	0.002	0.003	±	0.001	2.36	*
Allantoin	157.0362	0.323	±	0.042	0.845	±	0.119	2.62	**
UMP	323.0272	0.005	±	0.003	0.014	±	0.003	2.62	**
Ferulate	193.0507	0.001	±	0.001	0.002	±	0.002	2.92	
N-Acetyl-b-Ala	130.0505	0.000	±	0.001	0.001	±	0.001	3.49	
Citramalate	147.0294	0.007	±	0.006	0.026	±	0.016	3.54	*
Gluconate	195.0503	0.048	±	0.025	0.172	±	0.089	3.57	**
Glycerate	105.0194	0.004	±	0.001	0.016	±	0.010	3.85	*
Galacturonate	193.0322	0.001	±	0.001	0.003	±	0.002	4.83	*
Hexanoate	115.0766	0.001	±	0.001	0.004	±	0.002	6.80	**
N-acetylSer	146.0459	0.001	±	0.001	0.004	±	0.001	7.40	**
PEA	140.0115	0.002	±	0.001	0.017	±	0.006	8.43	**
HMG	161.0449	0.002	±	0.001	0.017	±	0.003	9.12	**
Allantonate	175.0466	0.007	±	0.002	0.065	±	0.028	9.87	**
AcetylGlu	188.0555	0.002	±	0.000	0.020	±	0.003	11.36	**

[1.] Anion metabolites annotated and quantified are listed. Signal intensity is presented as mean ± SD ($n = 6$). Fold change is presented as the ratio of each metabolite in mature seeds of GT to the corresponding value in NT. [2.] Asterisks (* and **) indicate significant differences between NT and GT at $P < 0.05$ and $P < 0.01$, respectively, as determined by *t*-test.

Table S3. Changes in cation metabolite levels in mature seeds determined by CE-MS.

Annotation Name	Ave m/z	Signal intensity						Fold change	*P* value
		NT			GT				
Trigonelline	138.0518	0.009	±	0.005	0.001	±	0.002	0.12	**
GSH	308.0899	1.756	±	0.319	1.026	±	0.410	0.58	**
Glucosaminate	196.0806	0.047	±	0.030	0.033	±	0.010	0.72	
2-Aminoadipate	162.0754	0.006	±	0.005	0.006	±	0.007	0.97	
5-MethylTHF	460.2013	0.002	±	0.000	0.002	±	0.001	1.00	
Adenosine	268.1029	0.097	±	0.047	0.099	±	0.035	1.02	
Anthranilate	138.0541	0.590	±	0.090	0.668	±	0.104	1.13	
2-MethylSer	120.0650	0.030	±	0.004	0.040	±	0.005	1.31	**
5-Methylcytosine	126.0648	0.002	±	0.001	0.003	±	0.001	1.32	
Cytosine	112.0502	0.005	±	0.001	0.008	±	0.001	1.48	**
Betaine	118.0861	0.905	±	0.220	1.339	±	0.354	1.48	*
Stachydrine	144.1015	0.044	±	0.031	0.068	±	0.069	1.54	
Thiamine	265.1106	0.054	±	0.006	0.092	±	0.012	1.71	**
Carnitine	162.1114	0.007	±	0.002	0.012	±	0.010	1.73	
Imidazolw-4-acetate	127.0497	0.006	±	0.001	0.010	±	0.001	1.79	**
Adenine	136.0614	0.010	±	0.003	0.021	±	0.008	2.17	**
Cytidine	244.0917	0.006	±	0.001	0.016	±	0.002	2.57	**
Ophthalmate	290.1332	0.031	±	0.010	0.084	±	0.038	2.71	**
Pyridoxamine5P	249.0640	0.001	±	0.002	0.004	±	0.000	2.78	**
GSSG	613.1607	0.027	±	0.007	0.078	±	0.012	2.83	**
3-MethylHis	170.0912	0.023	±	0.007	0.066	±	0.008	2.86	**
HydroxyPro	132.0647	0.014	±	0.007	0.046	±	0.017	3.16	**
Nicotinate	124.0388	0.013	±	0.002	0.043	±	0.007	3.38	**
Citrulline	176.1025	0.003	±	0.005	0.011	±	0.013	3.46	
Nicotinamide;Isonicotinamide	123.0547	0.004	±	0.004	0.016	±	0.003	3.64	**
Pyridoxamine	169.0971	0.002	±	0.002	0.008	±	0.003	4.10	**
g-Guanidinobutyrate	146.0917	0.037	±	0.005	0.158	±	0.113	4.27	*
b-Ala	90.0551	0.041	±	0.013	0.180	±	0.076	4.38	**
Guanosine	284.0977	0.013	±	0.002	0.061	±	0.008	4.81	**
Choline	104.1071	0.798	±	0.288	4.743	±	1.121	5.94	**
Pipecolinate	130.0857	0.017	±	0.004	0.119	±	0.037	6.98	**
GABA	104.0703	0.158	±	0.067	1.138	±	0.419	7.19	**
2-Aminobutyrate	104.0702	0.011	±	0.009	0.094	±	0.006	8.38	**
Agmatine	131.1284	0.001	±	0.002	0.011	±	0.005	8.82	**
Guanine	152.0562	0.000	±	0.001	0.003	±	0.001	10.01	**
Serotonin	177.1015	0.003	±	0.004	0.035	±	0.030	11.26	*
Ornithine	133.0969	0.002	±	0.003	0.034	±	0.013	16.16	**
SAH	385.1276	0.000	±	0.001	0.009	±	0.003	40.50	**
Cys-GSSG	427.0944	0.000	±	0.001	0.051	±	0.023	170.81	**
Trp (dimer)	409.1919	0.002	±	0.002	10.012	±	1.376	6619.06	**

[1.] Anion metabolites annotated and quantified are listed. Signal intensity is presented as mean ± SD (n = 6). Fold change is presented as the ratio of each metabolite in mature seeds of GT to the corresponding value in NT. [2.] Asterisks (* and **) indicate significant differences between NT and GT at $P < 0.05$ and $P < 0.01$, respectively, as determined by t-test.

Table S4. Untargeted negatively charged metabolic profiles in mature seeds detected by LC-MS.

Peak number	Retention time (min)	Mass number (m/z)	Signal intensity[1]						Fold Change[2]
			NT			GT			
10057	4.23	1179	0.184	±	0.108	0.003	±	0.000	0.01
9610	3.01	1102	0.091	±	0.097	0.003	±	0.000	0.03
4793	3.49	614	0.077	±	0.048	0.003	±	0.000	0.04
8237	4.43	885	0.191	±	0.077	0.009	±	0.014	0.05
7993	3.08	856	0.106	±	0.104	0.005	±	0.006	0.05
7830	3.29	841	0.107	±	0.070	0.006	±	0.009	0.06
7685	4.90	827	0.099	±	0.061	0.006	±	0.008	0.06
8239	5.08	885	0.056	±	0.021	0.003	±	0.001	0.06
7975	2.32	855	0.111	±	0.084	0.007	±	0.010	0.06
8919	3.12	985	0.126	±	0.090	0.009	±	0.010	0.07
5508	2.11	664	0.140	±	0.079	0.010	±	0.005	0.07
9651	2.97	1109	0.135	±	0.091	0.013	±	0.012	0.09
2057	3.60	382	0.051	±	0.015	0.522	±	0.128	10.22
5233	2.09	647	0.007	±	0.005	0.072	±	0.019	10.37
4218	3.20	571	0.013	±	0.011	0.152	±	0.079	12.11
2974	2.76	472	0.009	±	0.014	0.123	±	0.036	13.95
3645	2.80	527	0.008	±	0.009	0.124	±	0.021	14.81
1135	4.53	283	0.003	±	0.000	0.065	±	0.011	21.20
3885	3.47	545	0.009	±	0.007	0.220	±	0.084	23.42
1870	3.52	365	0.006	±	0.007	0.143	±	0.051	24.89
5839	4.99	683	0.003	±	0.000	0.078	±	0.029	25.53
3829	3.96	541	0.003	±	0.001	0.092	±	0.036	27.07
4993	4.31	627	0.003	±	0.000	0.084	±	0.019	27.61
1389	4.96	316	0.004	±	0.001	0.110	±	0.010	30.91
3539	5.65	518	0.003	±	0.000	0.097	±	0.018	31.94
4023	4.93	554	0.003	±	0.000	0.100	±	0.029	32.83
7199	2.11	784	0.003	±	0.000	0.100	±	0.012	32.87
1238	4.60	297	0.003	±	0.000	0.100	±	0.025	32.88
8310	4.91	894	0.005	±	0.005	0.169	±	0.070	33.24
4752	2.69	611	0.003	±	0.000	0.103	±	0.021	33.73
8572	3.54	926	0.003	±	0.000	0.106	±	0.041	34.65
1270	4.50	302	0.003	±	0.000	0.106	±	0.018	34.67
1786	3.49	356	0.003	±	0.000	0.127	±	0.027	41.56
406	3.70	171	0.003	±	0.000	0.131	±	0.013	43.10
1364	4.64	314	0.003	±	0.000	0.133	±	0.020	43.53
3870	3.40	544	0.016	±	0.010	0.803	±	0.293	50.34
7277	4.14	791	0.003	±	0.000	0.162	±	0.032	53.22
4611	3.64	601	0.003	±	0.000	0.174	±	0.043	57.26
4874	3.23	619	0.003	±	0.000	0.182	±	0.049	59.72
3869	3.27	544	0.010	±	0.008	0.576	±	0.221	60.51
1362	3.85	314	0.009	±	0.004	0.552	±	0.061	61.22
603	5.01	204	0.003	±	0.000	0.191	±	0.155	62.75

Table S4. *Cont.*

Peak number	Retention time (min)	Mass number (m/z)	Signal intensity[1]						Fold Change[2]
			NT			GT			
7917	2.23	849	0.005	±	0.004	0.362	±	0.061	67.48
6100	4.26	700	0.003	±	0.000	0.206	±	0.075	67.71
1555	3.87	332	0.026	±	0.012	1.791	±	0.310	70.03
4021	4.70	554	0.003	±	0.000	0.250	±	0.072	82.16
9141	2.96	1019	0.004	±	0.002	0.389	±	0.040	88.41
5692	4.21	674	0.003	±	0.000	0.283	±	0.053	92.87
4209	3.64	570	0.003	±	0.000	0.315	±	0.177	103.37
7357	3.57	797	0.003	±	0.001	0.353	±	0.105	104.29
6329	3.12	716	0.003	±	0.000	0.357	±	0.147	117.21
1418	3.15	319	0.004	±	0.002	0.544	±	0.109	133.96
8868	3.34	977	0.003	±	0.000	0.489	±	0.161	160.47
2148	5.21	389	0.003	±	0.000	0.738	±	0.224	242.51
4022	4.82	554	0.003	±	0.000	0.973	±	0.283	319.60
2335	3.00	407	0.014	±	0.003	4.766	±	0.238	334.46
7907	2.16	848	0.003	±	0.000	1.259	±	0.229	391.38

[1]Signal intensity is presented as mean ± SD (n = 6). [2]Fold change is presented as the ratio of each peak in mature seeds of GT to the corresponding value in NT. Listed here are peaks with fold changes of over 10 or under 0.1 (significant difference between NT and GT at $P < 0.05$ as determined by t-test).

Table S5. Untargeted positively charged metabolic profiles in mature seeds detected by LC-MS.

Peak number	Retention time (min)	Mass number (m/z)	Signal intensity[1]						Fold Change[2]
			NT			GT			
10529	5.33	1019	0.094	±	0.071	0.002	±	0.000	0.02
8588	4.30	798	0.056	±	0.033	0.002	±	0.000	0.03
9307	3.08	858	0.056	±	0.048	0.003	±	0.003	0.06
6125	4.06	653	0.035	±	0.008	0.002	±	0.000	0.06
1733	4.79	321	0.011	±	0.005	0.149	±	0.020	13.88
6834	2.02	692	0.003	±	0.002	0.058	±	0.013	17.47
3594	2.82	461	0.005	±	0.002	0.092	±	0.014	20.25
661	4.93	204	0.008	±	0.008	0.167	±	0.105	21.28
784	3.70	217	0.034	±	0.019	0.721	±	0.044	21.47
4242	5.12	520	0.002	±	0.000	0.042	±	0.009	21.79
1940	3.89	334	0.103	±	0.061	2.374	±	0.257	23.10
3426	2.68	447	0.002	±	0.000	0.051	±	0.011	26.41
858	2.72	225	0.003	±	0.002	0.073	±	0.009	27.18
5917	5.08	642	0.002	±	0.000	0.053	±	0.010	27.50
1594	3.32	307	0.002	±	0.000	0.053	±	0.009	27.50
4688	4.38	557	0.002	±	0.000	0.056	±	0.016	28.73
4734	4.12	561	0.002	±	0.000	0.065	±	0.012	33.66
10711	4.74	1046	0.003	±	0.002	0.095	±	0.024	33.86
1732	4.52	321	0.002	±	0.000	0.070	±	0.011	34.01*
5491	3.71	614	0.002	±	0.000	0.072	±	0.027	37.07
2872	5.26	395	0.002	±	0.000	0.072	±	0.015	37.11

Table S5. *Cont.*

Peak number	Retention time (min)	Mass number (m/z)	Signal intensity[1] NT			GT			Fold Change[2]
1678	4.66	316	0.002	±	0.000	0.074	±	0.010	38.11
6707	4.99	685	0.002	±	0.000	0.077	±	0.027	39.57
4671	4.50	556	0.002	±	0.000	0.082	±	0.020	42.53
2440	3.28	366	0.002	±	0.000	0.087	±	0.037	44.77
5074	2.60	588	0.003	±	0.002	0.149	±	0.061	51.43
4243	5.66	520	0.002	±	0.000	0.103	±	0.020	53.10
4384	3.44	532	0.002	±	0.000	0.117	±	0.044	60.20
8353	3.84	779	0.008	±	0.009	0.491	±	0.152	62.29
6212	3.98	659	0.002	±	0.000	0.153	±	0.028	79.10
7049	4.27	702	0.002	±	0.000	0.161	±	0.059	83.24
5277	3.66	603	0.002	±	0.000	0.173	±	0.049	89.16
3619	4.01	463	0.002	±	0.000	0.180	±	0.032	93.08
3023	4.60	407	0.002	±	0.001	0.233	±	0.088	98.44
4344	2.92	529	0.002	±	0.000	0.204	±	0.021	105.28
4674	4.93	556	0.002	±	0.000	0.220	±	0.073	113.82
10255	3.35	979	0.002	±	0.000	0.223	±	0.084	115.26
4889	3.66	573	0.002	±	0.000	0.227	±	0.129	117.09
4343	2.85	529	0.002	±	0.000	0.231	±	0.026	119.32
2926	3.57	400	0.002	±	0.000	0.241	±	0.062	124.62
1722	3.73	320	0.002	±	0.001	0.301	±	0.053	133.45
6206	2.28	659	0.002	±	0.000	0.262	±	0.114	135.33
6194	3.42	658	0.002	±	0.000	0.285	±	0.104	147.30
1305	3.48	277	0.002	±	0.000	0.299	±	0.042	154.50
10552	3.11	1022	0.002	±	0.000	0.316	±	0.024	163.37
2262	4.19	354	0.002	±	0.000	0.317	±	0.050	163.93
2456	2.99	367	0.002	±	0.000	0.324	±	0.123	167.55
3043	3.17	410	0.005	±	0.004	0.779	±	0.051	171.98
4672	4.70	556	0.002	±	0.000	0.339	±	0.117	174.97
3925	3.63	493	0.002	±	0.000	0.387	±	0.106	199.64
8838	3.02	818	0.002	±	0.000	0.508	±	0.037	262.60
2817	5.23	391	0.003	±	0.003	1.044	±	0.234	336.45
4870	3.61	572	0.002	±	0.000	0.667	±	0.357	344.61
4673	4.82	556	0.003	±	0.004	1.371	±	0.410	397.02
9212	2.17	850	0.002	±	0.000	0.800	±	0.243	412.99

Details as in Table S4. An asterisk (*) in the Fold change row indicates annotation as 5'-deoxy-5'-methylthioadenosine
